# Reduction of amputations of frostbitten limbs by treatment using microwave rewarming

**DOI:** 10.1038/s41598-023-28535-x

**Published:** 2023-01-24

**Authors:** Grigory Dunaevskiy, Eugene Gavrilin, Alexander Pomytkin, Roman Sorokin, Artem Kuznetsov, Vladimir Antipov, Aleksander Nechaev

**Affiliations:** 1 National Research Tomsk State University, Tomsk, 634050 Russia; 2 Tomsk Medical Sanitary Unit No. 2, Tomsk, 634050 Russia; 3 Tomsk Municipal Hospital No. 3, Tomsk, 634050 Russia

**Keywords:** Diseases, Medical research, Risk factors

## Abstract

Drug treatment of limb frostbite injuries is complicated due to the poor delivery of medications to affected tissues. External rewarming of the frostbitten area is risky and does not always result in positive outcomes because the dilatation of superficial vessels with constricted deep vessels can lead to irreversible damage, necrosis, and amputation. One of the techniques to restore perfusion of deep vessels in an affected extremity is rewarming with low-power microwave radiation in a specially designed metal chamber. Below are findings following treatment of 14 volunteers with this technique in 2 Tomsk hospitals during winters of 2018–2021. It is demonstrated that timely, i.e. in the early reactive period, application of microwave radiation and appropriate supportive drug treatment results in positive amputation-free outcomes. The key requirement is prompt thermal insulation of the trauma and no prior exposure to external heat sources.

## Introduction

Deep frostbites of extremities remain a serious problem not only in the Arctic and Antarctic but also other cold areas and countries, high-altitude settlements, stations, and resorts. The conventional external rewarming with deep vessels being ischemic can cause thrombosis and necrosis. At the same time, cold-induced injuries, often resulting in amputation, are mostly managed by conservative methods^1–3^ and no equipment exists to treat this condition.

In severe frostbites, one of the major problems of administration of vasodilators is that the actual delivery of the medications to affected tissues is complicated because of cold-induced constriction of vessels^4,5^.

One of the possible approaches to dilate deep blood vessels in frostbitten limbs is the use of low-power microwave radiation^6–9^. This method consists in using a metal microwave chamber where the affected limb is exposed to slow deep rewarming with electromagnetic radiation penetrating the frostbitten tissue and reaching deep blood vessels. The rewarming throughout the whole tissue depth triggers simultaneous activation of metabolic processes, blood flow, and lymph circulation both on the outside and inside the limb tissues and, consequently, prevent the aforementioned negative outcomes.

In general, blood and lymph circulation stops at a tissue temperature below + 15 °C, for hand digits the critical temperature is + 19 °C, for toes + 15 °C^5,10^.

Therefore, to prevent negative scenario of rewarming of a frostbitten limb, it is sufficient to rewarm it to approximately + 20–25 °C, after which the spontaneous recovery of life processes in the affected part and the delivery of supportive medications can be achieved. Thus, rewarming requires relatively low temperatures but it is critical that the entire volume rather than only outer areas should be rewarmed.

In many countries, current treatment protocols allow microwave electromagnetic waves to induce hyperthermia in malignant lesions^12,13^. For this purpose, powerful electromagnetic sources are used and temperatures in tumor and surrounding tissues exceed + 40 °C due to exposure to the electromagnetic field. In our case, such extensive heating is unnecessary.

Microwave radiation is also known to be used for physiotherapeutic procedures^14,15^, where the affected body organ is rewarmed by applying an antenna^16,17^. These devices are effective for local rewarming of both superficial tissue layers and deep layers located at a depth of a few centimeters. However, these methods cannot be employed in frostbitten extremities because they are aimed at local rewarming and do not ensure uniform exposure to heat of all the affected tissue layers.

The long-term use of the aforementioned hyperthermia-inducing and physiotherapeutic microwave devices in medical practice answers multiple questions regarding safety and absence of adverse effects, such as toxicity and carcinogenicity, particularly for low amplitudes and low integral absorbed dose.

The devices were installed in two Tomsk hospitals—Tomsk Medical Sanitary Unit No. 2 and Tomsk Municipal Hospital No. 3 during winters of 2018–2022.

The key condition was the absence of prior rewarming using other methods and thermal insulation of the cold-injured limb before microwave rewarming. Depending on the injury degree, the patients underwent 1–3 procedures. Thus, low-power exposure of the affected limbs only enabled in-depth rewarming of frostbitten tissues to temperatures that are necessary to restore the hemodynamics in deep blood vessels.

In the management of limb frostbites, in addition to microwave rewarming, both hospitals used standard sanitation procedures, pharmacological treatment and, if needed, fasciotomy and onychectomy.

## Results

The volunteers who were asked to participate in the study were patients with frostbites admitted to these hospitals for emergency care. The condition of these patients at admission and following treatment is documented in their medical histories and summarized in Tables 1 and 2.

**Table 1:** The outcomes of treatment of cold-induced limb injuries with microwave rewarming in MSU No. 2.

Patient (random number)	Age, gender	Affected limb	Frostbite degree	Comorbidity	ICD code	No. of procedures/microwave power (W)/time (min)	Treatment outcome
2–1	40–45, m	Right hand, left hand, right foot, left foot	3 3 2 2	Acute pancreatitis, Delirium tremens	T.35.4	2/45/30 2/45/30 2/45/30 2/45/30	Fingernails removed
2–2	25–30, m	Right foot, left foot	3 3	Delirium tremens	T.35.4	3/30/30 3/30/30	No amputation
2–3	40–45, m	Right hand, left hand,	4 4	HIV Hepatitis	T.35.4	1/15/30 1/15/30	No amputation
2–4	25–30, m	Right hand, left hand, right foot, left foot	3–4 3–4 3–4 3–4	2nd degree frostbite of lumbar region	T.35.4	2/30/30 2/30/30 2/60/30 2/60/30	Amputation of distal phalanges on toes 1–4 on both feet
**2–5**	30–35, m	Fingers, toes	1–3 1–3	HIV Hepatitis C	T.35.4	1/30/15 1/30/15 1/30/15 1/30/15	No amputation

**Table 2:** Outcomes of frostbite treatment with microwave rewarming in MH No. 3.

Patient (random number)	Age, gender	Affected limb	Frostbite degree	Comorbidity	ICD code	No. of procedures/microwave power (W)/time (min)	Treatment outcome
3–1	50–55, m	Right hand, left hand	2–3 2–3	IHD, angina pectoris, stage III hypertension	T.33.5	3/60/30 3/60/30	No amputation Superficial dry necrosis of distal phalanges of 3–4 fingers
3–2	85–90, m	All fingers on right hand, all fingers on left hand	3–4 3–4	IHD, paroxysmal AFib, dyslipidemia, dementia	T.35.4	3/60/30 3/60/30	Discharged without amputation with outpatient follow-up
3–3	65–70, m	All fingers on right hand, all toes on right foot	2–3 3–4	Mild anemia	T.35.4	3/60/30 3/60/30	All fingers and toes salvaged, superficial dry necrosis of distal phalanges of 2–3 fingers/toes
3–4	45–50, m	Fingers 2, 3 on right hand, fingers 2, 3 on left hand	4, 2	Chronic HVC, remission	T.35.4	3/60/30 3/60/30	Superficial dry gangrene of distal and medial phalanges of fingers 2, 3 on the right hand. After one month, a scheduled amputation of distal phalanges of fingers 2, 3 on the right hand. The main parts of the fingers saved
3–5	25–30, m	All fingers on right hand, all fingers on left hand	3, 3	None	T.35.4	3/60/30 3/60/30	All fingers salvaged. Discharged without amputation with outpatient follow-up
3–6	35–40, m	Right hand, left hand, right foot, left foot	3–4 3–4 2, 2	Chronic HVC	T.35.6	3/60/30 3/60/30 3/60/30 3/60/30	Discharged with outpatient follow-up. After one month, a scheduled amputation of distal phalanges of fingers. The main parts of the fingers saved
3–7	30–35, m	All fingers on right hand, all fingers on left hand	2–3	Mild anemia	T.34.5	3/60/30 3/60/30	All fingers salvaged. Cyanosis of distal phalanx tip of finger 3
3–8	20–25, m	All toes on right foot, toes 1, 2 on left foot, fingers 2, 3, 4 on right hand	1–2 1–2 2–3	Hypothyroidism, congenital tricuspid regurgitation	T.35.0	3/60/30 3/60/30 3/60/30	All fingers salvaged Discharged without amputation with outpatient follow-up
3–9	55–60, m	Toes on right foot	3–4	Stage III hypertension	T.35.1	3/60/30	Amputation of toes on right foot

Table 1 shows outcomes of treatment of cold-induced limb injuries with the microwave technique in Tomsk Medical Sanitary Unit No. 2 (MSU No. 2).

In all the listed patients, skin blisters appearing in the reactive period were opened, aspirated, and covered with a sterile bandage. The patients received standard drug therapy, consisting in IV administration of solutions warmed up to 40 °C, in the amount of up to 800–1000 ml to prevent edema and secondary microcirculation dysfunction. The drug therapy included antiaggregant, anticoagulant, antihypoxic, and antispasmodic therapy, as well agents aimed at improving microcirculation and hemorheology. In two patients, surgical removal of the gangrenous tissues was needed: in the first case (patient 2–1), the fingernails were removed, in the second (patient 2–4), distal phalanges on toes 1–4 on both feet were removed (this case report is published in detail^11^), but on the whole, the limbs were salvaged.

Table 2 shows outcomes of treatment of cold-induced limb injuries with microwave rewarming in 9 patients in Tomsk Municipal Hospital No. 3 (MH No. 3), 8 of whom arrived at the hospital with no prior rewarming of the affected limb and 1 patient (3–9) after an unsupervised attempt of rewarming.

This hospital employed the following comprehensive treatment strategy for frostbites: in the pre-reactive period, within the first hours on admission, all the patients underwent fasciotomy of affected limb segments considering compartment syndrome, onychectomy with thermally insulating bandages. Immediately after that, the first microwave rewarming procedure was performed.

Starting Day 1 after warming, the patients were daily administered intra-arterial (in femoral and brachial arteries) injections of 0.5% Novocaine to provide nerve blocks, anticoagulant, antiaggregant, and antispasmodic drugs to treat angiospasms and regenerate affected tissues (one procedure daily, on average 5–7 procedures in total). Simultaneously, the patients received IV drugs to improve blood circulation (Vasaprostan, Rheopolyglucin, Trental). Starting Days 2–3, physiotherapy procedures (magnetic therapy, 8–10 procedures) were administered to improve blood flow. To prevent infections, broad spectrum antibiotics (third-generation cephalosporins) were indicated, which were then adjusted based on sensitivity of wound cultures. The treatment measures also included laser blood irradiation with the Mulatto device^19^ 1.0–2.0 mW through the cubital vein (7–10 procedures).

In both hospitals, all patients were men over 20 years old. However, MSU No. 2 investigated the possibility of varying the duration and power of microwave warming, MH No. 3 examined the efficacy of identical exposure sessions.

All patients in both hospitals who received microwave treatment in the early reactive period demonstrated a positive outcome, with amputations of distal phalanges in some cases and no major amputations whatsoever.

With late admission, patient No. 3–9 in Tomsk Municipal Hospital No. 3, was admitted after 3 days of suffering a frostbite injury), necrosis of the toes of the affected limb could not be prevented despite combination treatment (pharmacological, surgical, laser, and microwave). Once necrosis and demarcation areas formed, doctors performed only reasonable debridement and skin grafting, making every effort to keep as much tissue of the limb area as possible and minimize the amputation scale and disability level.

## Discussion

The patients had different degrees of limb injuries (see the tables). For frostbites of first–second degree of severity, saving the entire limb is not difficult normally, and the effects of microwave rewarming are not so notable. For more complicated cases (third–fourth degree of severity), the entire limb, including digits/toes, cannot be saved using only surgical and pharmacological treatment. However, it is often the case when the severity of an injury and complications is evaluated inaccurately due to delayed manifestations of severe frostbite (the degree of frostbite in the tables is indicated as adjusted at the end of treatment). Thus, microwave rewarming can be recommended with any severity degree.

It is important that in the given cases, positive outcomes were demonstrated when, first, microwave rewarming had been used in the early reactive period, and, second, warming of the upper skin layers of the affected limb prior to microwave rewarming was minimized.

It should be noted that all volunteers in the study were only male subjects. With women and children, who have inherently less “volume” of the extremities, microwave power and procedure duration need to be reduced. It is reasonable to conduct a larger study considering the probability of other comorbidities.

The efficiency studies of frostbite microwave treatment in informed volunteers demonstrate the possibility of recovering from deep-level frostbites without major amputations. The treatment consisted in the microwave frequency of 2.45 GHz permitted for medical use and microwave power, which is less than the one used in physiotherapy procedures. The most efficient procedure was the combination of microwave rewarming and a surgical procedure (fasciotomy, onychectomy), as well as hemorheological drugs and vasodilators.

The findings suggest that although the number of patients who have received treatment for cold trauma using microwave warming is still small, the proposed method of reducing the volume of amputations seems promising.

## Methods

Prior approval of the Tomsk State University Committee of Bioethics was obtained to involve informed volunteers in this study (Meeting Minutes No. 1 dated November 23, 2018).

All experiments were performed in accordance with relevant named guidelines and regulations. Written informed consent to participate in the studies and to publish identifying information was obtained from all participants and/or their legal guardians.

**Figure 1:**
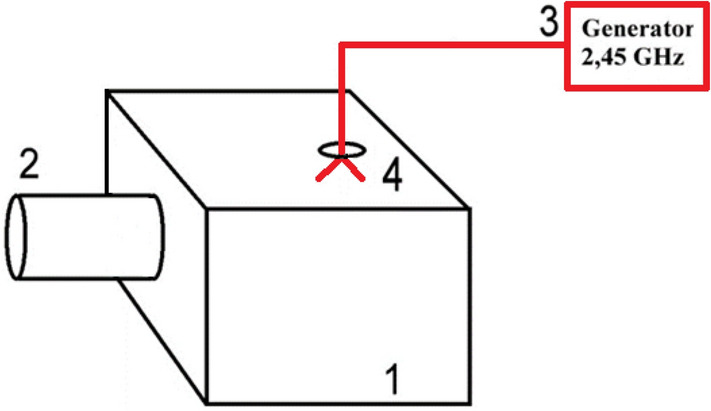
Design of the apparatus for rewarming limb frostbites. (1) A chamber. (2) A soft radioprotective sleeve. (3) A microwave generator. (4) An antenna.

This study suggests using microwave rewarming in a closed metal chamber^6,7,9^ to treat cold-induced injuries. The general design is shown in Fig. 1.

**Figure 2:**
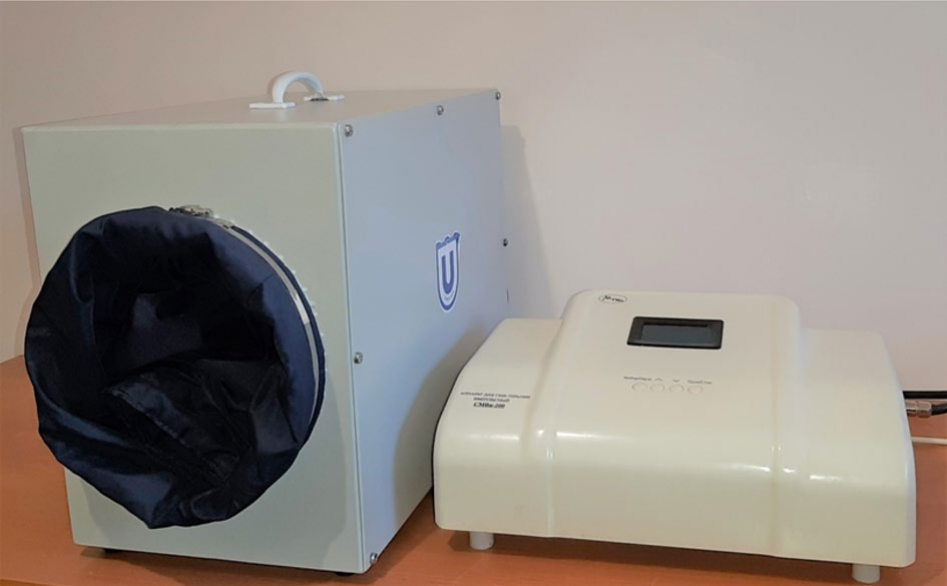
General view of the apparatus for rewarming limb frostbites.

The appearance of the chamber and generator is shown in Fig. 2.

The extremity is placed inside a chamber (1) through the opening equipped with a radioprotective metal sleeve (2). That helps protect a patient and healthcare personnel from the negative microwave radiation. The 2.45 GHz generating unit of a SMVI‑200 physiotherapeutic device^18^ is used as a source of microwave radiation (3). The excitation is performed by a built-in oscillator (antenna) (4), which is attached from the outside of the chamber to the generating unit with a cable. The generating unit of a SMVI‑200 ensures appropriate power output and duration of a rewarming session. The power reserve of this device is 200 watts, but only 60 watts were used to re-warm the frostbitten limb, and the actual heating power was about 30–40 watts, taking into account the partial reflection of waves from the camera. The duration of the session was 30 min. As the limb warmed up, the patients felt a slight warmth, hence, this method is not likely to be associated with discomfort. The usual painful feelings after warming up are not caused by microwave exposure.

## Data Availability

The datasets used and analyzed in this study are available from the corresponding author, G. Dunaevskiy, upon reasonable request.
